# Epidemiologic comparison of ankle injuries presenting to US emergency departments versus high school and collegiate athletic training settings

**DOI:** 10.1186/s40621-018-0163-x

**Published:** 2018-09-03

**Authors:** Alexandria J. Wiersma, Lina Brou, Sarah K. Fields, R. Dawn Comstock, Zachary Y. Kerr

**Affiliations:** 1 Children’s Hospital Colorado, 13123 E 16th Ave, Aurora, CO USA; 2 Section of Emergency Medicine, Department of Pediatrics, University of Colorado School of Medicine, 13123 East 16th Avenue, Box B251, Aurora, 80045 CO USA; 3 Eugene S. Farley Jr Health Policy Center, Department of Family Medicine, University of Colorado School of Medicine, Academic Office Building 1, Suite 4311, Aurora, 80045 CO USA; 4 Department of Communication, University of Colorado Denver, Campus Box 176, Denver, 80217 CO USA; 5 Colorado School of Public Health, University of Colorado Anschutz, Mailstop B119 13001 East 17th Place, Aurora, USA; 6 Program for Injury Prevention, Education, and Research (PIPER), Department of Epidemiology, University of Colorado, Building 500, W3145, Aurora, CO 80045 USA; 7 Department of Exercise and Sport Science, University of North Carolina, 313 Woollen Gym, CB# 8605, Chapel Hill, NC 27599 USA

**Keywords:** Ankle, Sports, Injury surveillance, Pediatrics, Collegiate athletes, Emergency department

## Abstract

**Background:**

Ankle sprains account for a large proportion of injuries presenting to both United States (US) emergency departments (EDs) as well as high school (HS) and collegiate school athletic training settings. The epidemiologic differences across these settings by both sport and diagnosis have not been well differentiated.

Ankle injury data from 3 national surveillance datasets. Athletic training setting data from the National High School Sports-Related Injury Surveillance System and the National Collegiate Athletic Association Injury Surveillance Program was from academic years 2009/10 through 2013/14 and the US Consumer Product Safety Commission’s National Electronic Injury Surveillance System (ED setting) data was from calendar years 2009 through 2013. Data was analyzed for patients 14–22 years old participating in 12 sports (male football, baseball, basketball, lacrosse, soccer, and wrestling, and female softball, basketball, lacrosse, soccer, volleyball, and field hockey). We calculated sport-specific injury rates, proportions, and rate ratios (RRs) with 95% confidence intervals (CI).

**Results:**

During the study period, the surveillance systems captured 20,261 ankle injuries presenting to EDs plus 5546 HS and 2725 collegiate injuries presenting to school athletic training settings. Rates were higher in collegiate compared to HS athletes presenting in the athletic training setting. Football accounted for the largest proportion of ankle injuries presenting to HS (31.2%) and college (41.0%) athletic training settings; male basketball accounted for the largest proportion presenting to EDs among both HS (41.0%) and college (65.8%) aged patients. Sprains/strains accounted for over 80% of injuries in all three settings. Fractures accounted for a larger proportion of ankle injuries presenting to EDs (9.5%) compared to HS (3.8%) and college (0.8%) athletic training settings. There was no change in injury rates during the study period across the three settings.

**Conclusions:**

Injury rates and patterns varied by sport and presentation setting, with athletic trainers evaluating more ankle injuries overall in the collegiate setting compared to the high school setting. Ankle injuries presenting to EDs were more commonly fractures, suggesting that more severe injuries present to this setting. Understanding the epidemiology of such patterns will help readers interpret differences in publications reporting data from varied clinical settings.

**Electronic supplementary material:**

The online version of this article (10.1186/s40621-018-0163-x) contains supplementary material, which is available to authorized users.

## Background

Prior studies have reported that sport-related ankle sprains account for large proportions of all injuries presenting to United States (US) Emergency Departments (EDs) and high school (HS) and college athletic trainers (ATs) in school settings (Waterman et al. 2010; Browne and Barnett 2016; Hootman et al. 2007; Rosa et al. 2014; Yard et al. 2008; Kerr et al. 2017; Fong et al. 2007; Dick et al. 2007a; Dick et al. 2007b; Agel et al. 2007a; Agel et al. 2007b; Agel et al. 2007c; Lanese et al. 1990; James et al. 2014; Putukian et al. 2014). The rate of ankle injuries presenting to US EDs is reported as 2.15 per 1000 person-years (Waterman et al. 2010). ED studies reported ankle injuries represented 22% (Boyce and Quigley 2004) to 50% (Waterman et al. 2010; Browne and Barnett 2016) of all sports-related injuries. As ankle injuries frequently inhibit individuals’ ability to maintain physically active lifestyles, the high injury incidence poses both clinical and public health concerns (Tanen et al. 2014; Delahunt et al. 2018; Gerber et al. 1998).

Ankle injury rates vary by sport. Prior research has found sports with rapid changes of direction or possible athlete-athlete contact have high ankle injury risks. One meta-analysis of 144 manuscripts on ankle sprain in sports, which reported findings by broad categories of sport rather than by individual sport, reported “indoor + court sports” had the highest prevalence of ankle sprain at 12.2% followed by “field sports” at 11.3% (Doherty et al. 2014). A study of National Collegiate Athletic Association (NCAA) athletes reported in 10 of 15 sports ankle injuries represented > 10% of all injuries with ankle injuries representing > 20% of all injuries in men’s and women’s basketball, and women’s volleyball (Hootman et al. 2007). More specifically, a study of NCAA Division 1 athletes found 27% of all reported musculoskeletal injuries were ankle/foot injuries, with the highest incidence in women’s gymnastics, women’s cross-country, women’s soccer and men’s cross country (Hunt et al. 2016). A different study evaluating ankle sprain in NCAA athletes, found rates highest for men’s football, wrestling, and ice hockey (Mauntel et al. 2017). A study of 20 HS sports reported the highest rates of ankle injury were in boys’ and girls’ basketball and gymnastics (Beynnon et al. 2005).

The epidemiology of ankle injuries varies according to where injured athletes seek care as well as across athlete subgroups. A recent study that evaluated all soccer injuries across multiple settings and age groups (school ATs and EDs in both HS and collegiate athletes) demonstrated that more severe injuries, with longer recovery times, presented to the ED setting (Kerr et al. 2017). Because physicians are not consistently available for practices and competitions across multiple sports in high school and collegiate settings in the US, ATs frequently have primary responsibility for injury prevention and reduction of further injury as the personnel on site charged with ensuring sports injuries are recognized early, treated immediately, and managed to enable proper healing (Lyznicki et al. 1999). Because ATs evaluate and provide care for minor injuries on site and refer more severe injuries to EDs or other clinical settings, patterns of injury presenting to different clinical settings may vary. Ankle injury rates and patterns also vary across other factors include athlete age and sex. Several studies have reported prevalence and incidence of ankle sprains are higher in collegiate athletic than high school athletes (Doherty et al. 2014) and higher in females than males (Tanen et al. 2014; Doherty et al. 2014; Swenson et al. 2013; Hosea et al. 2000; Kerr et al. 2016). However, other studies have failed to observe sex differences (Yard et al. 2008; Mauntel et al. 2017; Beynnon et al. 2005; Clark and Tanner 2003).

Understanding the epidemiology of ankle injuries across various clinical settings may help clinicians better recognize and manage ankle injuries most likely to present to their clinical setting, thus reducing the potential for chronic ankle problems (Tanen et al. 2014). Additionally, understanding differences in ankle injury rates and patterns across population sub-groups such as sport, gender, and age should help drive the development of more effective, population-specific injury prevention efforts. To date, it appears no published studies have provided this data through direct comparison of the epidemiology of ankle injuries across a variety of sports presenting to US school athletic training settings versus to EDs, or across large numbers of male and female sports by athlete age group (i.e. HS versus college). This study fills this gap in the literature by building on multiple prior studies that look specifically at one sport or age group. Our objectives were (1) to expand on this prior knowledge by comparing ankle injury rates and patterns presenting to US EDs and US HS and collegiate athletic training setting and (2) to evaluate population sub-group differences by comparing rates and patterns of ankle injuries by sport and gender.

## Methods

### Surveillance systems from which data was obtained

The US Consumer Product Safety Commission’s National Electronic Injury Surveillance System (NEISS), previously described, (US Consumer Product Safety Commission 2000) captures data on injuries related to consumer products and sports activities treated in a nationally representative sample of US EDs. NEISS receives data from approximately 100 US hospitals with 6 or more beds and 24-h EDs representing a stratified probability sample of the over 6000 US hospitals (including urban, suburban, rural, and children’s hospitals). Participating hospitals have trained NEISS coders who review ED charts extracting data for all injuries including the following variables of interest in this study: patient’s age, sex, injury diagnosis, injured body part, and product involved (which provides sport-specific product codes) Data from individual injury case records are weighted to produce national estimates.

The National High School Sports-Related Injury Surveillance System, High School Reporting Information Online (HS RIO) captures data from a large national sample of US high schools using two previously described concurrent surveillance cohorts (Schroeder et al. 2015). In brief, US high schools with a National Athletic Trainers’ Association-affiliated certified AT were eligible. In the original study cohort, participants were categorized into 8 strata based on US Census geographic region (United States Census Bureau 2000) and school population (enrollment ≤1000 or > 1000). Participants were randomly selected from each stratum to achieve a sample of 100 schools reporting for each of 9 sports included in the original HS RIO study (boys’ football, boys’ and girls’ soccer, girls’ volleyball, boys’ and girls’ basketball, boys’ wrestling, boys’ baseball, and girls’ softball). For the additional 13 sports added since 2008/2009, schools offering any of the new sports (girls’ field hockey, girls’ gymnastics, boys’ ice hockey, boys’ and girls’ lacrosse, boys’ and girls’ track & field, boys’ and girls’ swimming & diving, boys’ volleyball, boys’ and girls’ cross country, and cheerleading) were included in the expansion of the HS RIO study. Schools in either the original or expanded study were allowed to report for more than their assigned sports. Combined, these methodologies resulted in a large, nationally disperse convenience sample of US high schools reporting data on 22 sports. In this study, all data reported by the randomly selected sample and expanded sample were combined to create the study dataset. Participating ATs reported injury and athlete exposure (AE) data weekly throughout the academic year and were able to view and update previously submitted information as needed during the course of a season. Reportable injuries (1) occurred as a result of participation in a school sanctioned practice or competition, (2) required medical attention by an AT or physician, and (3) resulted in restriction of the student-athlete’s participation for one or more days post injury or were a concussion, dental injury, heat event, or fracture regardless of time loss. An AE was defined as one athlete participating in one school sanctioned practice or competition.

The NCAA Injury Surveillance Program (NCAA-ISP) is an Internet-based surveillance system that depends on a convenience sample of teams with ATs voluntarily reporting injury and exposure data. The NCAA-ISP has been previously described in detail (Kerr et al. 2014). In brief, the NCAA-ISP relies on a convenience sample of NCAA institutions. The NCAA institutions could elect to provide data for all sports or a sample of sports. Thus, participation varied by sport and year. During the 2009/10–2013/14 academic years, a common data element (CDE) standard allowed data to be gathered from different electronic health record/injury documentation applications, including the Athletic Trainer System (ATS [Keffer Development, Grove City, PA]), Injury Surveillance Tool (IST [Datalys Center, Indianapolis, IN]), and the Sports Injury Monitoring System (SIMS [FlanTech, Iowa City, IA]) (Kerr et al. 2014). The CDE export standard allowed ATs to document injuries as they normally would as part of their daily clinical practice, as opposed to asking them to report injuries solely for purposes of participation in an injury surveillance program. Reportable injuries were defined as: (1) occuring as a result of participation in an organized practice or competition; and (2) requiring medical attention by an AT or physician. The NCAA-ISP included all injuries regardless of time loss, and multiple injuries that occurred from one injury event. For each injury event, ATs from participating programs completed a detailed event report on the injury or condition (e.g., site, diagnosis) and the circumstances [e.g., activity, mechanism, event type (i.e., competition or practice)]. The ATs were able to view and update previously submitted information as needed during the course of a season. An AE was defined as one athlete participating in one school sanctioned practice or competition. These data were stripped of any identifiers and personally identifiable information, retained only relevant variables and values, and was examined by data quality control staff and a verification engine (Additional file 1).

### Data analyzed

We reviewed all ankle injuries sustained by14–22 year old athletes participating recreationally (NEISS) or for their school (HS RIO and NCAA-ISP). We studied twelve sports of interest (SOI), each of which were included in all three surveillance systems: male American football [hereinafter football] (NEISS code 1211), baseball (5041), basketball (1205), lacrosse (1215), soccer (1267), and wrestling (1270) and female softball (5034), basketball (1205), lacrosse (1215), soccer (1267), volleyball (1266), and field hockey (1295). The study period of interest was the five years from January 1, 2009 through December 31, 2013 for the calendar year based NEISS and 2009/10 through 2013/14 for the academic year based HS RIO and NCAA-ISP. The total number of ankle injuries captured during the study period was *n* = 20,261for NEISS, *n* = 5546 for HS RIO, and *n* = 2725 for NCAA-ISP. Primary variables of interest available for analysis in all three surveillance systems included sport played, diagnoses of injury, and year injured. Injury diagnoses were categorized as contusion, dislocation, fracture, laceration, sprain/strain, and “other.”

### Analyses

We analyzed the data using SAS 9.4 (SAS Institute, Cary, NC). We calculated sport-specific injury rates for NEISS data using case counts as the numerator and intercensal population estimates as the denominator. Because NEISS has a complex survey design based on a nationally representative probability sample of hospitals, all analyses accounted for statistical weighting and hospital stratum using specified variables by CSPC and survey procedures in SAS for analysis. Sport-specific injury rates for both HS RIO and NCAA-ISP were calculated using case counts as the numerator and AEs as the denominator. Trend significance for ankle injury rates over time for the five-year study period were analyzed using linear regression to create best-fit lines with *p*-values presented to evaluate statistical significance of slopes of best-fit lines. Rate Ratios (RR) with 95% Confidence Intervals (CI) were presented to compare sport-specific injury rates between HS RIO and NCAA-ISP. Additionally, sport and diagnosis differences between HS RIO and NEISS and between NCAA-ISP and NEISS were evaluated using injury proportion ratios (IPRs). All proportions are presented with 95% CIs. All *p*-values < 0.05 and proportions with non-overlapping 95% CIs were considered statistically significant. The institutional review board of Nationwide Children’s Hospital and the Research Review Board of the NCAA approved the study.

## Results

### Overall incidence and rates

During the study period, NEISS captured 20,261 ankle injuries presenting to US EDs among the 12 SOI, representing an estimated 712,162 injuries nationwide (Table 1). The overall rate of injuries presenting to the ED setting was 18.3 per 10,000 population with 66% of injuries among individuals 14–17 years old (HS aged) and 44% among individuals 18–22 years old (college aged). Males sustained 78% of injuries overall; 72% of HS age group injuries and 88.4% of college age group injuries. Males had significantly higher injury rates (27.6 per 10,000) than females (8.5 per 10,000) (RR: 3.3; 95% CI: 3.2, 3.3). Male basketball comprises the largest proportion of injuries (49.8%) (Table 1). Other sports with large proportions of injuries included male football (15.8%), female basketball (8.4%), male soccer (7.9%), and female soccer (6.3%) (Table 1).

**Table 1:** Ankle injuries sustained by 14–22 year olds participating in football, baseball, softball, basketball, lacrosse, soccer, volleyball, field hockey, or wrestling treated in US emergency departments from 2009 through 2013

Variable	Actual Count		National Estimates^a^	
	n	%	n	%
Sex				
Male	15,799	78.0	551,665	77.5
Female	4462	22.0	160,497	22.5
Sport				
Football	3204	15.8	111,169	15.6
Baseball	564	2.8	22,318	3.1
Softball	616	3.0	24,775	3.5
Male Basketball	10,084	49.8	351,710	49.4
Female Basketball	1707	8.4	58,313	8.2
Male Lacrosse	119	0.6	4093	0.6
Female Lacrosse	100	0.5	3389	0.5
Male Soccer	1598	7.9	53,404	7.5
Female Soccer	1267	6.3	45,850	6.4
Volleyball	733	3.6	27,087	3.8
Field Hockey	39	0.2	1083	0.2
Wrestling	230	1.1	8971	1.3
Diagnosis				
Contusion	346	1.7	13,526	1.9
Dislocation	63	0.3	1850	0.3
Fracture	1920	9.5	65,008	9.1
Laceration	29	0.1	1063	0.1
Sprain/Strain	16,955	83.7	588,507	82.6
Other	948	4.7	42,208	5.9
Disposition				
Released	19,977	98.6	704,089	98.9
Hospitalized	152	0.8	3631	0.5
Other	132	0.7	4442	0.6
Total	20,261	100.0	712,162	100.0

^a^National estimates were calculated by applying statistical weights provided by the US Consumer Product Safety Commissions’ National Electronic Injury Surveillance System to actual case counts

HS RIO and NCAA-ISP captured 5546 and 2725 ankle injuries, respectively, sustained by HS and collegiate student-athletes participating in the 12 SOI (Table 2). Among HS athletes, sports with the highest injury rates included girls’ soccer (5.2 per 10,000 AE) girls’ basketball (5.1), boys’ basketball (5.0), and football (4.7). Among college athletes, sports with the highest injury rates were men’s basketball (16.2 per 10,000 AE), women’s soccer (13.9), men’s soccer (13.0), football (12.4), and women’s basketball (12.2). Rates per 10,000 were higher overall in the college setting.

**Table 2:** Rates of ankle injuries by sport and level of play, high school (HS RIO) and college (NCAA-ISP), during the 2009/10 through 2013/14 academic years

Sport	HS RIO	Rate per 10,000 AE	NCAA-ISP	Rate per 10,000 AE
	# of Ankle Injuries		# of Ankle Injuries	
Football	1733	4.7	1116	12.4
Baseball	120	0.9	54	3.0
Softball	185	2.0	55	3.4
Male Basketball	847	5.0	350	16.2
Female Basketball	693	5.1	238	12.2
Male Lacrosse	116	2.1	114	7.1
Female Lacrosse	107	2.7	88	8.3
Male Soccer	404	2.9	207	13.0
Female Soccer	615	5.2	300	13.9
Volleyball	480	3.7	127	8.1
Field Hockey	79	1.7	19	5.1
Wrestling	167	1.4	57	7.2

### Distribution by sport and diagnosis

Football accounted for the largest proportion of all injuries (31.2% and 41.0%, respectively) in the HS RIO and NCAA-ISP datasets, followed by boys’/men’s basketball (15.3% and 12.8%, respectively) (Table 3). Sprains/strains represented nearly all diagnoses among HS (92.2%) and college (89.3%) aged student-athletes. Fractures represented 3.8% of HS injuries, but only 0.8% of collegiate injuries (Table 3).

Male basketball accounted for nearly half of all injuries in the NEISS dataset (Table 1), accounting for the highest proportion of injuries among HS (41.0% of all injuries, 95% CI: 37.9–44.1) and college (65.8%, 95% CI: 63.7–67.9) aged individuals (Table 3). The most common ED diagnosis was sprain/strain (83.7%); however, fractures represented 9.5% of all injuries. Nearly all individuals (98.6%) presenting to the ED with ankle injuries were treated and released (Table 1).

**Table 3:** Comparison of proportions of ankle injuries by sport and diagnosis among individuals treated in US emergency departments from 2009 through 2013 (NEISS) and seen by athletic trainers in high school (HS RIO) and collegiate (NCAA-ISP) settings during the 2009/10–2013/14 academic years

Variable	14–17 year olds (High School Age Range)			18–22 year olds (College Age Range)		
	HS RIO	NEISS	IPR^a^	NCAA-ISP	NEISS	IPR^c^
	% (95% CI)	% (95% CI)		% (95% CI)	% (95% CI)	
Sport						
Football	31.2 (30.0–32.5)	18.0 (16.3–19.6)	1.7^b^	41.0 (39.1–42.8)	11.0 (9.7–12.3)	3.6^d^
Baseball	2.2 (1.8–2.5)	3.5 (2.9–4.0)	0.6^b^	2.0 (1.5–2.5)	2.5 (2.0–3.0)	0.8
Softball	3.3 (2.9–3.8)	4.3 (3.7–5.0)	0.8	2.0 (1.5–2.5)	1.8 (1.2–2.4)	1.1
Male Basketball	15.3 (14.3–16.2)	41.0 (37.9–44.1)	0.4^b^	12.8 (11.6–14.1)	65.8 (63.7–67.9)	0.2^d^
Female Basketball	12.5 (11.6–13.4)	9.9 (9.0–10.9)	1.3^b^	8.7 (7.7–9.8)	4.8 (4.1–5.5)	1.8^d^
Male Lacrosse	2.1 (1.7–2.5)	0.7 (0.4–0.9)	3.0^b^	4.2 (3.4–4.9)	0.4 (0.2–0.6)	10.4^d^
Female Lacrosse	1.9 (1.6–2.3)	0.6 (0.3–0.9)	3.2^b^	3.2 (2.6–3.9)	0.2 (0.0–0.3)	18.8^d^
Male Soccer	7.3 (6.6–8.0)	7.2 (6.1–8.2)	1.0	7.6 (6.6–8.6)	8.1 (7.0–9.3)	0.9
Female Soccer	11.1 (10.3–11.9)	8.2 (6.8–9.6)	1.4^b^	11.0 (9.8–12.2)	2.9 (2.3–3.6)	3.7^d^
Volleyball	8.7 (7.9–9.4)	4.8 (4.0–5.6)	1.8^b^	4.7 (3.9–5.5)	1.8 (1.4–2.3)	2.6^d^
Field Hockey	1.4 (1.1–1.7)	0.2 (0.1–0.4)	7.0^b^	0.7 (0.4–1.0)	0.0 (0.0–0.0)	56.8^d^
Wrestling	3.0 (2.6–3.5)	1.6 (1.3–1.9)	1.9^b^	2.1 (1.6–2.6)	0.6 (0.3–0.9)	3.6^d^
Diagnosis						
Contusion	1.8 (1.4–2.1)	2.2 (1.9–2.6)	0.8	4.6 (3.8–5.4)	2.4 (2.0–2.8)	1.9^d^
Dislocation	0.3 (0.1–0.4)	0.2 (0.1–0.3)	1.5	0.1 (0.0–0.3)	0.3 (0.2–0.4)	0.3
Fracture	3.8 (3.3–4.3)	9.7 (8.6–10.8)	0.4^b^	0.8 (0.5–1.1)	9.3 (8.3–10.3)	0.1^d^
Laceration	0.0 (0.0–0.0)	0.1 (0.0–0.2)	0.0	0.1 (0.0–0.2)	1.4 (1.1–1.6)	0.1^d^
Sprain/Strain	92.2 (90.7–93.6)	81.8 (76.5–87.1)	1.1^b^	89.3 (88.2–90.5)	80.4 (75.5–85.2)	1.1^d^
Other	2.0 (1.0–3.1)	6.0 (0.3–11.7)	0.3	5.0 (4.2–5.8)	6.2 (1.2–11.3)	0.8

^a^Compares HS RIO to NEISS

^b^indicates no overlap between 95% confidence intervals

^c^Compares NCAA-ISP to NEISS

^d^indicates no overlap between 95% confidence intervals

Differences in injury patterns by sport appeared across both age groups. A higher proportion of injuries presenting to the HS athletic training setting were sustained by football players (31.2%) compared to HS aged athletes presenting to the ED (18.0%; IPR = 1.7) (Table 3). A similar result existed among college aged athletes (41.0% vs. 11.0%; IPR = 3.6). A lower proportion of ankle injuries presenting to the HS athletic training setting were sustained by boys’ basketball players (15.3%) compared to HS aged athletes presenting to the ED (41.0%; IPR = 0.4). College aged athletes had similar results (12.8% vs. 65.8%; IPR = 0.2). In HS RIO and NCAA-ISP, basketball ankle injury rates had similar results those of other sports; however, in NEISS, basketball rates were higher compared other sports (Table 1).

A higher proportion of ankle injuries presenting to the HS athletic training setting were sprains/strains (92.2%) compared to HS aged athletes presenting to the ED (81.8%; IPR = 1.1). College aged athletes had similar results (89.3% vs. 80.4%; IPR = 1.1). A lower proportion of ankle injuries presenting to the HS athletic training setting were fractures (3.8%) compared to HS aged athletes presenting to the ED (9.7%; IPR = 0.4). College aged athletes had similar results (0.8% vs. 9.3%; IPR = 0.1).

**Fig. 1:**
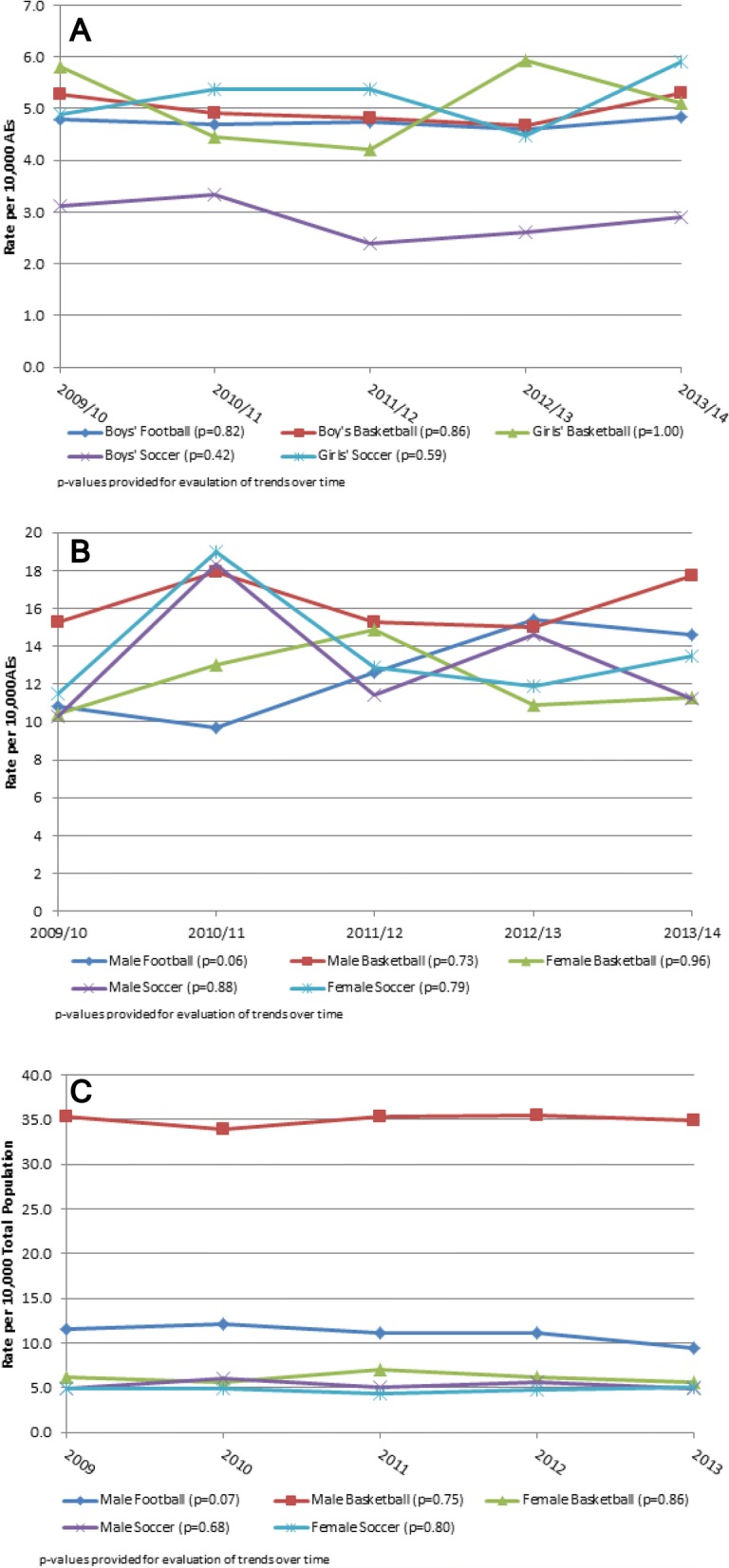
**a** Rates of ankle injuries per 10,000 athletic exposures seen by athletic trainers in the high school athletic training setting. **b** rates of ankle injuries per 10,000 athletic exposures seen by athletic trainers in the collegiate athletic training setting. **c** rates of ankle injuries among individuals treated in United States emergency departmentss per 10,000 total population

The five sports with the highest injury rates demonstrated no significant trends over time in injury rates among individuals presenting to the HS athletic training setting (Fig. 1a), the college athletic training setting (Fig. 1b), or the ED (Fig. 1c).

**Fig. 2:**
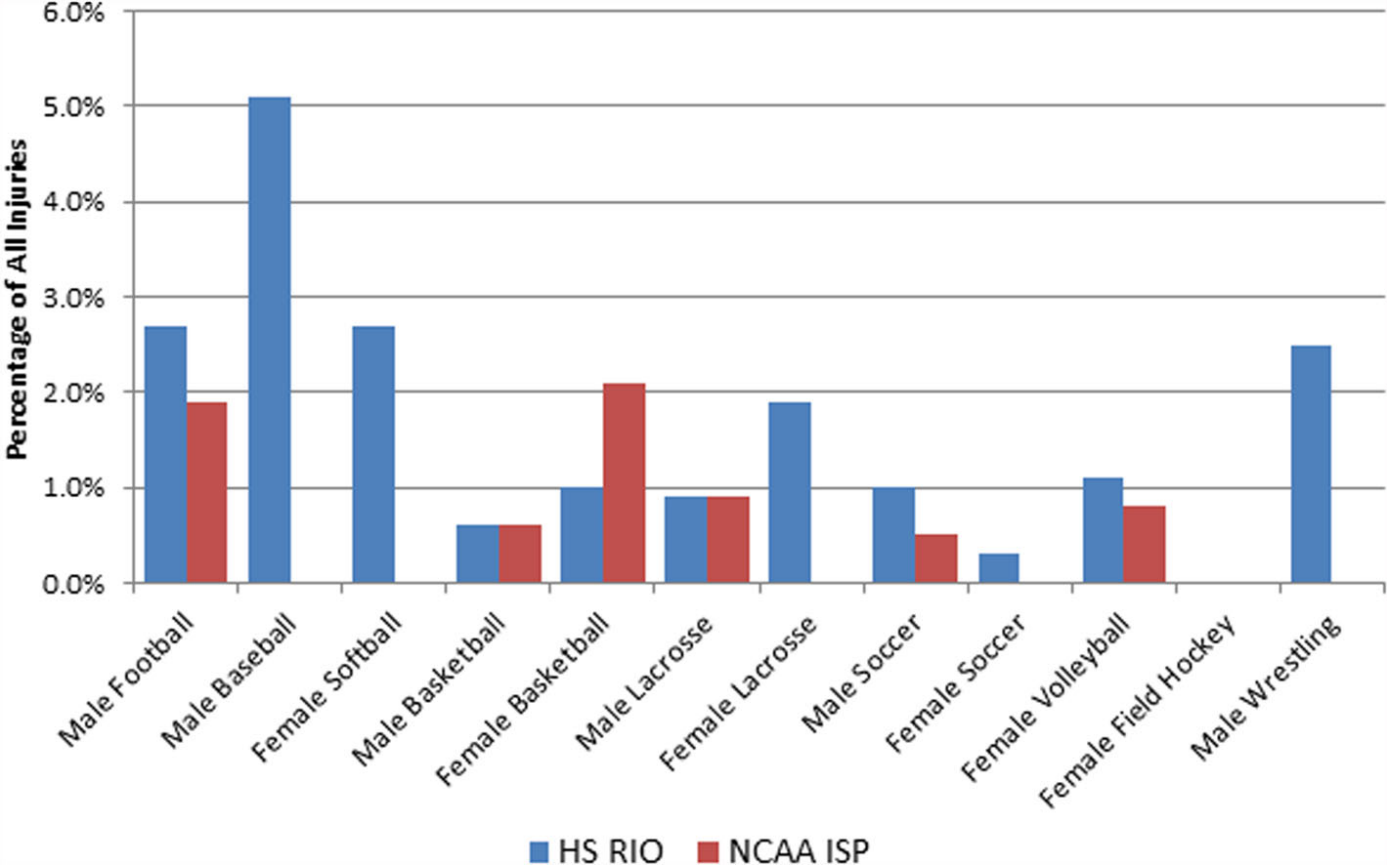
Percentage of all ankle injuries resulting in surgical repair by sport and level of play

Patterns of surgical intervention varied by sport and age group (Fig. 2). For HS aged athletes, boys’ baseball had the highest proportion of injuries resulting in surgical intervention (5.1%), followed by girls’ softball (2.7%), boys’ football (2.7%), and boys’ wrestling (2.5%). A lower proportion of ankle injuries sustained by college aged athletes resulted in surgical intervention with the highest proportion in women’s basketball (2.1%) followed by men’s football (1.9%) and male lacrosse (0.9%).

## Discussion

Sports-related ankle injuries are common (Browne and Barnett 2016; Hootman et al. 2007; Rosa et al. 2014; Yard et al. 2008; Kerr et al. 2017; Fong et al. 2007; Dick et al. 2007a; Dick et al. 2007b; Agel et al. 2007a; Agel et al. 2007b; Agel et al. 2007c; Lanese et al. 1990; James et al. 2014; Putukian et al. 2014; Boyce and Quigley 2004). Evaluating ankle injuries captured by three large national surveillance systems, we found injury patterns varied across sports, age groups, and clinical settings. Despite the clinical attention afforded these common sports injuries, among the five sports in this study with the highest ankle injury rates, no significant trends over time existed among individuals presenting to the HS athletic training setting, college athletic training setting, or ED setting during the five year study period. There were higher rates of injury in the collegiate setting (Table 2) compared to the HS setting and injuries presenting to ED settings were more severe (e.g., more fractures) than those presenting to school athletic training settings. Understanding the epidemiology of ankle injuries across various clinical settings may help clinicians better recognize and manage ankle injuries most likely to present to their clinical setting, thus reducing the potential for chronic ankle problems (Tanen et al. 2014). Additionally, understanding differences in ankle injury rates and patterns across population sub-groups such as sport, gender, and age should help drive the development of more effective, population-specific injury prevention efforts. Thus, recognizing differences across clinical settings and population sub-groups is important.

Many previous ankle injury studies have focused on sprains (Waterman et al. 2010; Gerber et al. 1998; Doherty et al. 2014; Beynnon et al. 2005). Consistent with these prior reports, sprains/strains were the most common diagnosis in this study. However, diagnoses varied by presentation setting with fractures representing a significantly higher proportion of injuries presenting to EDs then athletic training settings in both age groups (9.7% vs 3.8% among high school age athletes and 9.3% vs 0.8% among college age athletes). This suggests that more severe injuries present to the ED while less severe injuries are either referred elsewhere (e.g., sports med clinic, ortho clinic, etc) or managed in the school athletic training setting. Our findings are consistent with prior reports (Kerr et al. 2017). Although injury diagnosis patterns were quite similar across HS and collegiate aged athletes presenting to the ED setting, fractures represented a greater proportion of injuries presenting to the HS athletic training setting than the collegiate athletic training setting. Additionally, in each sport but female basketball a greater proportion of ankle injuries sustained by HS athletes resulted in surgical intervention compared to collegiate athletes. This may be due to the inclusion of injuries resulting in time loss under 24 h in the NCAA-ISP. Nevertheless, understanding such variations will help readers interpret differences in publications reporting data from different clinical settings.

Doherty et al. found “indoor + court sports” had higher ankle injury rates than “field sports” (Doherty et al. 2014). We found ankle injury rates in basketball were similar to rates in soccer and football in both the HS and collegiate athletic training settings; in the ED setting, male basketball comprised the largest proportion of injuries. However, similar to a previous study of NCAA athletes which showed football had the highest number of ankle injuries, (Hootman et al. 2007), we found football accounted for the highest proportion of all ankle injuries presenting to both the HS and collegiate athletic training settings. The causes of these sport differences across clinical settings is unclear and warrants further investigation. One potential explanation for our findings is that in school based sports football has much larger rosters than basketball (The National Federation of State High School Associations 2013–14), whereas recreational basketball is more commonly played than football because basketball can be played year round (both indoors and outside) with fewer players and minimal protective equipment.

Using the NEISS data set to evaluate sports-related injury rates is complicated by the lack of athletic exposure data in this data set, or any reliable US population-based sport specific athlete exposure data. In this study we calculated rates for NEISS data using case counts as the numerator and intercensal population estimates as the denominator. Although this was the best available denominator data, interpretation of these rates should be made with caution. Acknowledging this important limitation, ankle injury rates in the ED setting in this study were higher among males than females in most gender comparable sports, contradicting some previous studies (Tanen et al. 2014; Doherty et al. 2014; Swenson et al. 2013; Hosea et al. 2000; Kerr et al. 2016) but consistent with others (Yard et al. 2008; Gerber et al. 1998; Mauntel et al. 2017; Beynnon et al. 2005; Clark and Tanner 2003). This difference may be due to the large number of male basketball injuries, which likely helped drive the high injury rate among males. No significant trends were identified in rates of ankle injury over time in athletes presenting to the HS athletic training setting, the college athletic training setting, or the ED setting. Given the high incidence of sports-related ankle injuries coupled with the potential for chronic ankle problems resulting from ankle injury (Delahunt et al. 2018; van Rijn et al. 2008), increased efforts are needed to decrease ankle injury rates. Evidence-based injury prevention programs should be more widely adopted. For example, despite recent randomized control trials conducted by McGuine et al. demonstrating lace-up ankle braces reduced acute ankle injuries among high school basketball and football players, both with and without a previous history of ankle injury (McGuine et al. 2011; McGuine et al. 2012), widespread adoption of use of lace-up ankle braces has not yet occurred. A better understanding of injury patterns across population subgroups and clinical settings should help drive the development, implementation, and evaluation of more effective, targeted prevention programs.

These findings highlighted above clearly demonstrate that the epidemiology of ankle injuries differs across various clinical settings. Although the patient populations of these different healthcare settings have some overlap, the patient populations also differ. For example, large numbers of high school and collegiate aged recreational athletes do not have access to school based ATs and some athletes participating in school sanctioned sports do not have access to ATs (e.g., the AT may be covering a different sport at the time of an athlete’s injury, the high school may not employ an AT, etc.). These populations are more likely to present only to an ED or some other health care setting (e.g., sports medicine clinic, etc.). Conversely, ATs triage injuries and, in consultation with physicians, frequently manage more minor injuries in the high and collegiate school athletic training settings. Thus, the populations presenting to school based athletic training settings and ED settings may actually have little overlap, particularly for less serious injuries. Recognizing differences across clinical settings is necessary to gain a full understanding of the epidemiology of ankle injuries among high school and collegiate athletes.

The limitations of this study are associated with the limitations of the three national surveillance studies. For example, accurate national sports participation data is difficult to obtain. While this limitation necessitated our utilization of intercensal population estimates to calculate rates for the NEISS data, this method likely resulted in an underestimation of the true rates of sports-related ankle injuries presenting to the ED setting. The NEISS dataset also lacked follow-up detail provided in the HS RIO and NCAA-ISP datasets such as return to play time and need for surgery. However, whereas NEISS captures all sports-related ankle injuries presenting to EDs from all types of sports and recreational activities, HS RIO and the NCAA-ISP only capture injuries sustained during school-sanctioned sports activities. Additionally, HS RIO is limited to high schools with an AT and the NCAA-ISP is limited to NCAA institutions; the generalizability of our findings to all HS and college athletes may be limited. Although we found several significant epidemiologic differences in ankle injuries presenting to athletic training versus ED settings, injured athletes first presenting to athletic training settings may subsequently be referred to EDs; thus an over count of ankle injuries, particularly more severe injuries (e.g. fractures), may have occurred. Similarly, the difference between the academic year time-frame for HS RIO and NCAA-ISP and the calendar year time frame for NEISS could lead to either over or under counts in injuries presenting to EDs although this concern is lessened given the lack of significant ankle injury rate trends over time across the three settings. Despite these limitations, this study makes an important and novel contribution, providing the first direct comparison of ankle injury rates and patterns across sports, age groups, and clinical settings.

## Conclusion

Sports-related ankle injuries are common and may be cared for by different types of providers in different clinical settings, especially ATs in school athletic training settings and ED physicians. Variation in rates and patterns by age, sex, sport, clinical setting and injury severity were identified in this study. Understanding such differences will help readers interpret other published reports. Our findings also highlight the higher rates of injury in collegiate athletes as well the increased severity of injuries presenting to the ED setting. Understanding such patterns will remind clinicians (either AT or physician) to be more vigilant for the injuries most likely to present to their clinical setting. Our findings also highlight the importance of sports injury surveillance programs to continue to facilitate the effort to drive more effective population-specific prevention strategies.

## Additional file

Additional file 1: **Table S1.** Description of national injury surveillance datasets analyzed in the study. (DOCX 96 kb)

## Abbreviations

AE: Athletic exposure
AT: Athletic trainers
CDE: Common data element
CI: Confidence intervals
ED: Emergency Departments
HS RIO: High School Reporting Information Online
HS: High School
IPR: Injury proportion ratios
NCAA: National Collegiate Athletic Association
NCAA-ISP: NCAA Injury Surveillance Program
NEISS: National Electronic Injury Surveillance System
RR: Rate ratios
SOI: Sports of interest
US: United States

## References

[CR1] Agel J, Olson D, Dick R (2007). Descriptive epidemiology of collegiate Women’s basketball injuries: National Collegiate Athletic Association Injury Surveillance System, 1988-1989 through 2003-2004. J Athl Train.

[CR2] Agel J, Palmieri-Smith R, Dick R (2007). Descriptive epidemiology of collegiate Women’s volleyball injuries: National Collegiate Athletic Association Injury Surveillance System, 1988-1989 through 2003-2004. J Athl Train.

[CR3] Agel J, Ransone J, Dick R (2007). Descriptive epidemiology of collegiate Men’s wrestling injuries: National Collegiate Athletic Association Injury Surveillance System, 1988-1989 through 2003-2004. J Athl Train.

[CR4] Beynnon B, Vacek P, Murphy D (2005). First-time inversion ankle ligament trauma. The effects of sex, level of competitions and sport on the incidence of injury. Am J Sports Med.

[CR5] Boyce S, Quigley M (2004). Review of sports injuries presenting to an accident and emergency department. Emerg Med J.

[CR6] Browne G, Barnett P (2016). Common sports-related musculoskeletal injuries presenting to the emergency department. J Paediatr Child Health.

[CR7] Clark K, Tanner S (2003). Evaluation of the Ottawa ankle rules in children. Pediatr Emerg Care.

[CR8] Delahunt E, Bleakley CM, Bossard DS, et al. Clinical assessment of acute lateral ankle sprain injuries (ROAST): 2019 consensus statement and recommendations of the International Ankle Consortium. Br J Sports Med. 2018; 10.1136/bjsports-2017-098885. [Epub ahead of print] 10.1136/bjsports-2017-098885 29886432

[CR9] Dick R, Ferrara M, Agel J (2007). Descriptive epidemiology of collegiate Men’s football injuries: National Collegiate Athletic Association Injury Surveillance System, 1988-1989 through 2003-2004. J Athl Train.

[CR10] Dick R, Hertel J, Agel J (2007). Descriptive epidemiology of collegiate Men’s basketball injuries: National Collegiate Athletic Association Injury Surveillance System, 1988-1989 through 2003-2004. J Athl Train.

[CR11] Doherty C, Delahunt E, Caulfield B (2014). The incidence and prevalence of ankle sprain injury: a systematic review and meta-analysis of prospective Epidemiogical studies. Sports Med.

[CR12] Fong D, Hong Y, Chan L (2007). A systematic review on ankle injury and ankle sprain in sports. Sports Med.

[CR13] Gerber J, Williams G, Scoville C (1998). Persistent disability associated with ankle sprains: a prospective examination of an athletic population. Foot Ankle Int.

[CR14] Hootman J, Dick R, Agel J (2007). Epidemiology of collegiate injuries for 15 sports: summary and recommendations for injury prevention initiatives. J Athl Train.

[CR15] Hosea T, Carey C, Harrer M (2000). The gender issue: epidemiology of ankle injuries in athletes who participate in basketball. Clin Orthop Relat Res.

[CR16] Hunt K, Hurwit D, Robell K (2016). Incidence and epidemiology of foot and ankle injuries in elite collegiate athletes. Am J Sports Med.

[CR17] James L, Kelly V, Beckman E (2014). Injury risk management plan for volleyball athletes. Sports Med.

[CR18] Kerr Z, Dompier T, Snook E (2014). National collegiate athletic association injury surveillance system: review of methods for 2004-2005 through 2013-2014 data collection. J Athl Train.

[CR19] Kerr Z, Kroshus E, Grant J (2016). Epidemiology of Nathional Colelgiate athletic association Men’s and Women’s cross-country injuries, 2009-2010 through 2013-2014. J Athl Train.

[CR20] Kerr Z, Pierpoint L, Currie D (2017). Epidemiologic comparisons of soccer related injuries presenting to emergency departments and reported within high school and collegiate settings. Inj Epidemiol.

[CR21] Lanese R, Strauss R, Leizman D (1990). Injury and disability in matched Men’s and Women’s intercollegiate sports. Am J Public Health.

[CR22] Lyznicki JM, Riggs JA, Champion HC (1999). Certified athletic trainers in secondary schools: report of the council on scientific affairs, american medical association. J Athl Train.

[CR23] Mauntel T, Wikstrom E, Roos K (2017). The epidemiology of high ankle sprains in National Collegiate Athletic Association Sports. Am J Sport Med.

[CR24] McGuine TA, Brooks A, Hetzel S (2011). The effect of lace-up ankle braces on injury rates in high school basketball players. Am J Sports Med.

[CR25] McGuine TA, Hetzel S, Wilson J, Brooks A (2012). The effect of lace-up ankle braces on injury rates in high school football players. Am J Sports Med.

[CR26] Putukian M, Lincoln A, Crisco J (2014). Sports-specific issues in Men’s and Women’s lacrosse. Curr Sports Med Rep.

[CR27] Rosa B, Asperti A, Helito C (2014). Epidemiology of sports injuries on collegiate athletes at a single center. Acta Ortop Bras.

[CR28] Schroeder A, Comstock R, Collins C (2015). Epidemiology of overuse injuries among high-school athletes in the United States. J Peds.

[CR29] Swenson D, Collins C, Fields S (2013). Epidemiology of US high school sports-related ligamentous ankle injuries, 2005/06-2010/11. Clin J Sport Med.

[CR30] Tanen L, Doherty C, Van Der Pol B (2014). Prevalence of chronic ankle instability in high school and division I athletes. Foot Ankle Spec.

[CR31] The National Federation of State High School Associations. 2013–14 High School Athletics Participation Survey. http://www.nfhs.org/ParticipationStatics/PDF/2013-14_Participation_Survey_PDF.pdf. Accessed 12 June 2018.

[CR32] United States Census Bureau (2000). Census Regions of the United States.

[CR33] US Consumer Product Safety Commission (2000). NEISS: National Electronic Injury Surveillance System, A Tool for Researchers.

[CR34] van Rijn R, van Os A, Bernse R (2008). What is the clinical course of acute ankle sprain? A systematic literature review. Am J Med.

[CR35] Waterman B, Owens B, Davey S (2010). The epidemiology of ankle sprain in the United States. J Bone Joint Surg Am.

[CR36] Yard E, Schroeder M, Fields S (2008). The epidemiology of United States high school soccer injuries, 2005-2007. Am J Sports Med.

