# Supplementary material for: Epidemiologic comparison of ankle injuries presenting to US emergency departments versus high school and collegiate athletic training settings
Source: Inj Epidemiol. 2018 Sep 3;5:33. doi: 10.1186/s40621-018-0163-x (PMC6119677; doi:10.1186/s40621-018-0163-x)
Supplement: Supplementary file 1 — Table S1. Description of national injury surveillance datasets analyzed in the study. (DOCX 96 kb) [file 40621_2018_163_MOESM1_ESM.docx]

|  | **HS RIO** | **NCAA-ISP** | **NEISS** |
| --- | --- | --- | --- |
| Administered By | Dr. Dawn Comstock, Colorado School of Public Health | Datalys Center for Sports Injury Research and Prevention | United States Consumer Product Safety Commission |
| Website | http://www.ucdenver.edu/academics/colleges/PublicHealth/research/ResearchProjects/piper/projects/RIO/Pages/default.aspx | http://www.datalyscenter.org/ | https://www.cpsc.gov/Research--Statistics/NEISS-Injury-Data |
| Study Population | High athletes participating in sports in a national sample of US high schools | Collegiate athletes participating in sports a national sample of NCAA institutions | Individuals presenting for care of injuries in a national sample of US emergency departments |
| Injuries Captured by the Surveillance System | All time loss injuries sustained by student athletes in the sports under study and reported to the surveillance system by the school’s AT | All time loss injuries sustained by student athletes in the sports under study and reported to the surveillance system by the school’s AT | All injuries presenting for care (non-sports related as well as sports-related) and reported to the surveillance system by the NEISS ED chart abstractor |
| Surveillance System Data Analyzed in this Study | All ankle injuries presenting to HS RIO schools from 2009/10-2013/14 sustained by high school athletes participating in male football, baseball, basketball, lacrosse, soccer, and wrestling and female softball, basketball, lacrosse, soccer, volleyball, and field hockey; n=5,546 | All ankle injuries presenting to NCAA-ISP schools from 2009/10-2013/14 sustained by collegiate athletes participating in male football, baseball, basketball, lacrosse, soccer, and wrestling and female softball, basketball, lacrosse, soccer, volleyball, and field hockey; n=2,725 | All ankle injuries presenting to NEISS EDs from 2009-2013 coded as having been associated with male football (NEISS code 1211), baseball (5041), basketball (1205), lacrosse (1215), soccer (1267) and wrestling (1270) and female softball (5034), basketball (1205), lacrosse (1215), soccer (1267), volleyball (1266), and field hockey (1295); n=20,261 |
| Exclusion Criteria | 1. Any injury, other than an ankle injury, sustained by an athlete participating in any of the 12 sports of interest 2. Any ankle injury sustained by an athlete playing any other sport | 1. Any injury, other than an ankle injury, sustained by an athlete participating in any of the 12 sports of interest 2. Any ankle injury sustained by an athlete playing any other sport | 1. Any injury, other than ankle injury, associate with any of the 12 NEISS codes of interest 2. Any ankle injury associate with any other NEISS code |

**Table 1. Description of National Injury Surveillance Datasets Analyzed in the Study**
